# Outcomes, mechanisms and contextual factors of positive psychology interventions for health workers: a systematic review of global evidence

**DOI:** 10.1186/s12960-021-00564-5

**Published:** 2021-02-27

**Authors:** Maartje Kletter, Bronwyn Harris, Celia Brown

**Affiliations:** University of Warwick (WMS), Coventry, CV4 7AL UK

**Keywords:** Positive psychology, Health personnel, Systematic review, Intervention impact, Realist evaluation

## Abstract

**Background:**

Interventions using positive psychology (PP), which build on positive qualities of healthcare personnel and institutions, could potentially enhance organisational performance in healthcare. The aim of this systematic review was to identify if PP interventions have an impact on organisational performance of healthcare personnel, and if so, how this impact can be achieved. We developed a logic model to explain the impact of PP interventions on organisational performance.

**Methods:**

We searched Web of Science, Medline, Psychinfo, Embase, Scopus and CINAHL (from inception until March 2019) and references of included articles to identify studies that evaluated the impact of a PP intervention for health personnel. Study quality was assessed using the SQUIRE checklist for quality improvement studies. Data were extracted about study details, setting, participants, intervention, method of evaluation and results. Outcomes, mechanisms and contexts were coded in nVivo. Data synthesis was guided by Lewis’ theory of the impact of PP interventions on organisational performance and Kneale et al.’s method for logic model development. Collected data were integrated into a logic model explaining initial inputs, processes, and intermediate outcomes of PP interventions that lead to improved organisational performance in healthcare settings.

**Results:**

We retrieved 4638 articles and identified five through references of included articles of which 29 studies (31 articles) met our inclusion criteria. Most articles were of low quality (*n* = 19) and outcome measures varied widely. We identified 54 different outcomes of PP interventions, including ‘improved well-being’ and ‘improved interaction and support’. Forty-nine mechanisms were identified including ‘recognising and reframing negative interpretations’. Twenty four contextual factors were identified of which seven acted as barriers. ‘Managerial support’ was a facilitator mentioned in eight studies. All identified outcomes, mechanisms and contextual factors were integrated into a logic model explaining how interventions using PP can impact organisational performance in healthcare.

**Conclusion:**

Few identified outcomes were statistically significant, however, trends in both quantitative and qualitative outcomes show that PP interventions can increase well-being and interaction and support and thus improve organisational performance in healthcare. The developed logic model can be used in the implementation and evaluation of interventions using PP for health personnel.

## Introduction

Health systems worldwide face increased demand for care due to aging populations and growing prevalence of chronic diseases, alongside needing to deliver acute and preventive care [1]. On the supply-side, a lack of human resources [2] contributes to understaffed healthcare organisations. Particularly during the COVID-19 pandemic, healthcare personnel worldwide face enormous pressure due to heavy workloads and staff shortages, insufficient personal protective equipment (PPE) and high risk of infection [3]. Heavy workloads, job insecurity and concerns over personal safety (potentially created by a lack of physical resources such as PPE) [4] are job demands, the physical, social or organisational aspects of the job that require sustained physical or mental effort [5]. Increased job demands lead to mental health problems, including anxiety, depression, insomnia and burnout, a condition of emotional exhaustion, depersonalisation and a sense of low accomplishment [3, 6–8]. The negative effects of job demands can be mitigated by job resources, such as workplace social support, performance feedback, job control and personal growth and development, leading to increased well-being and decreased burnout [5, 9–12].

**Fig. 1:**
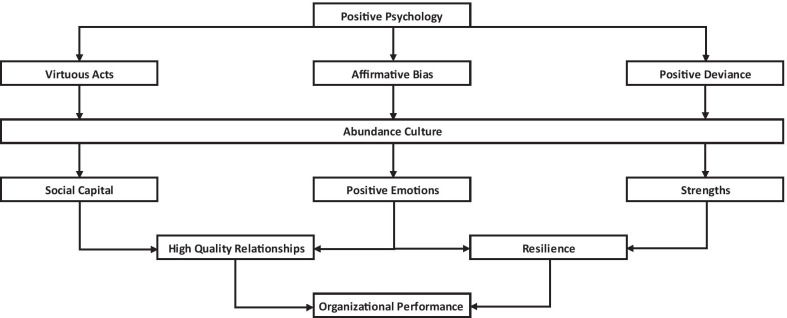
Theory of impact of positive psychology interventions on organisational performance by Lewis [16]

Job resources could potentially be improved with interventions based on positive psychology (PP). These interventions aim to drive change by building on positive qualities and strengths of healthcare personnel. PP focuses on three dimensions: positive emotions, positive traits and positive institutions, in which people flourish [13, 14]. PP interventions can potentially impact positively on organisational performance of healthcare workers as explained in the theory by Lewis, shown in Fig. 1 [15, 16].

A systematic review and meta-analysis looking at the impact of PP interventions at work showed that interventions using PP can improve desirable job outcomes like well-being and job engagement (*g* = 0.25, SE 0.04, *p* < 0.01, 95% CI 0.17–0.33). Additionally, PP interventions can reduce job stress (*g* = − 0.34, SE 0.12, *p* < 0.01, 95% CI − 0.57–− 0.11) [17]. Another literature review, summarising findings of PP interventions in organisational contexts, showed that PP interventions consistently enhanced employee well-being [18]. While PP interventions have been introduced for patients [19], the approach is not yet implemented widely for healthcare personnel. As part of a 2018 exploratory study, we found that existing PP interventions are welcomed by health personnel [20], however, in general, little is known about the impact of PP interventions on organisational performance in healthcare, about how this impact could be achieved and which contextual factors are important to take into account when designing and implementing a PP intervention in the specific setting of healthcare workplaces. In this study, we aimed to identify if and how PP interventions impact healthcare personnel and organisational performance in different healthcare settings and contexts. Through this systematic review—using realist evaluation methodology—we aimed to identify the outcomes (including effectiveness), mechanisms and contextual factors of interventions using PP for health personnel in global settings. We aimed to collate this evidence into a logic model to explain the impact of PP interventions on organisational performance in healthcare. By going beyond “is it effective?”, and including mechanisms and contextual factors in the logic model as well as outcomes, our work can support future designers, implementers and evaluators of PP interventions aimed at improving well-being and performance of healthcare personnel.

## Methods

A protocol of this systematic review was published on Prospero on 19-12-2018 (PROSPERO 2018 CRD42018120114) [21].

We searched Web of Science, Medline, Psychinfo, Embase, Scopus and CINAHL from inception to 03-01-2019. Additionally, we hand-searched the references of included articles for other eligible studies.

To identify articles regarding the impact of PP interventions for health workers, we used the following search terms: health workers AND (positive psychology OR appreciative inquiry (AI)). The latter term, and its components, were included as AI is a type of PP intervention that we may not have picked up with just PP. Additionally, we searched for other terms that may describe PP interventions without the term PP being used: strengths or strength-based in proximity of feedback or coaching, positive in the proximity of coaching or feedback and excellence in the proximity of feedback. The full search strategy can be found in Additional file 1: Search strategy.

### Study selection

Any study, of any design, that evaluated the impact of interventions informed by PP for any type of health worker (regardless of training, setting, gender or age), with any kind of outcome, was included. Interventions were determined to be informed by PP if they aimed to drive change or quality improvement based on positive qualities. If PP was described but not evaluated, or used as a research method only, the study was excluded. Studies that were not reported in English were also excluded.

Titles and abstracts were all screened by MK. Additionally CB and BH independently screened 100 randomly selected titles and abstracts, of which 25 were identical. Articles were randomly selected with the help of Excel random number generator, all articles were assigned a random number and the lowest 25 numbers were chosen for both CB and BH to screen. Two additional random numbers were assigned and again the 75 articles with the lowest numbers were assigned to CB and BH, respectively. Disagreements regarding inclusion of the articles, at both the title and abstract screening and in the full-text screening, performed by MK, were resolved through discussion with all authors.

### Data extraction

Extraction sheets were developed to extract information about the following: study details (i.e. year of publication, country, study design), setting, participants, intervention as per TiDIER checklist [22], method of evaluation, outcome measures and results. The TiDIER checklist was chosen for data extraction regarding the intervention as it guided us in collecting comparable components of PP interventions across studies. Our data synthesis was informed by realist evaluation methodology [23] which identifies outcomes (effects of the intervention), mechanisms (explaining processes through which outcomes are achieved) and contextual factors (conditions needed to trigger mechanisms that produce particular outcomes). Data were coded in nVivo, by MK. Only outcomes, mechanisms and contexts that were described as being present in the organisation in which the evaluation was implemented (i.e. not those hypothesised in the article) were coded. All data were extracted by MK, checked by CB and BH, and discussed during face-to-face meetings.

### Quality assessment

The quality of the included studies was assessed with the help of the SQUIRE checklist for quality improvement (QI) studies [24], as in healthcare settings PP is often intended for QI purposes. The SQUIRE checklist consists of five main categories. An included study was considered to be of high quality if out of the five SQUIRE checklist categories a maximum of two categories were considered to be of medium quality, with the other categories being of high quality. If three to five of the categories were considered to be of medium quality, with no category of low quality, the study was considered to be of medium quality. If one category was considered to be of low quality, and one category of medium quality, with the rest being of high quality, the study was considered to be of medium quality. In all other cases the study was considered to be of low quality. MK assessed the quality of the included studies and this was checked by CB and BH.

### Data analysis

Data were summarised for countries, interventions and type of health personnel/ward involved. Coded outcomes, mechanisms and contextual factors were grouped based on similarity in order to develop themes.

Due to differences in study designs and outcome measures, quantitative synthesis of quantitative outcomes was not possible. To enable synthesis of quantitative and qualitative outcomes together, identified outcomes were qualified as positive in quantitative findings if the assessed outcome showed statistically significant improvement and as positive in qualitative findings if an improvement in the outcome was described. Outcomes were qualified as neutral in quantitative findings if no statistically significant improvement or deterioration was present and as neutral in qualitative findings if no improvement or deterioration was described. Finally, outcomes were qualified as negative in quantitative outcomes if the outcome showed statistically significant deterioration and in qualitative outcomes if the outcome was described to deteriorate. Outcomes were clustered into larger groups based on similarity.

Mechanism themes were listed, including in which articles they were mentioned. Contextual themes, and in which articles they were mentioned were listed, as well as if they acted as facilitator or barrier for producing the intended outcome.

### Data synthesis

We used Kneale et al.’s step-based method logic model development to synthesise our data [25]. Kneale et al.’s method was chosen as it provides a structured way to develop a logic model, as part of a systematic review, which is what we aimed to do [25]. The logic model depicts a chain of components representing mechanisms and contextual factors leading to outcomes. Presenting results in a logic model could develop a shared understanding of processes and underlying mechanisms for programme implementers, designers or evaluators [25].

Step one of Kneale et al.’s method regards the examination of existing theory or logic models explaining how PP interventions can impact organisational performance, including potential mechanisms, contextual factors and intermediate outcomes. We identified the theory by Lewis [16], which explains that PP interventions create an abundance culture through three key mechanisms: virtuous acts, affirmative bias and positive deviance. Virtuous acts are acts undertaken regardless of reciprocity whereas in organisations with affirmative bias strengths and possibilities are emphasised. Positive deviance in an organisation means there is a focus on creating an affluence of good. These three mechanisms help create an abundance culture, which is an essential element of organisations with exceptional organisational performance [15, 16].

Within the abundance culture, social capital is created as people are attracted to virtuous actors. Additionally, an affluence of good supports the creation of positive emotions and enhanced strengths [15]. Positive emotions and enhanced strengths support the creation of resilience, as described by the broaden-and-build theory by Fredrickson [26], who proposes that when positive emotions are experienced and strengths are enhanced thought–action repertoires are broadened [27].

Step two of Kneale et al.’s method is the identification of the distal outcome, which for this study is organisational performance [25]. Having identified an existing theory to use as a starting point for our logic model and our distal outcome, we could then proceed with integrating the evidence from the studies included in our review with the existing theory. We did this by comparing the details of the theory with the empirical evidence base in the step-wise process advocated by Kneale et al. For step three and four, intermediate outcomes as used in the studies included in our review were identified. Step four entails the identification of intermediate outputs, which are the direct focus for modification within the activities of the intervention, but we did not distinguish these from outcomes [25].

Steps five and six are the identification of mechanisms and intervention inputs (contextual factors) in the studies, respectively. Lewis’ theory only includes mechanisms, explaining how the outcomes of a PP programme are achieved [15, 16]. Because contextual factors impact the mechanisms we planned to add these at the top of our logic model. We subcategorised contextual factors as follows [25, 28]: factors before design and implementation of the intervention (factors present in the organisation that support enthusiasm for interventions), factors during the design (factors that support uptake of the intervention) and factors during the intervention itself (factors that support effectiveness of the intervention).

## Results

**Fig. 2:**
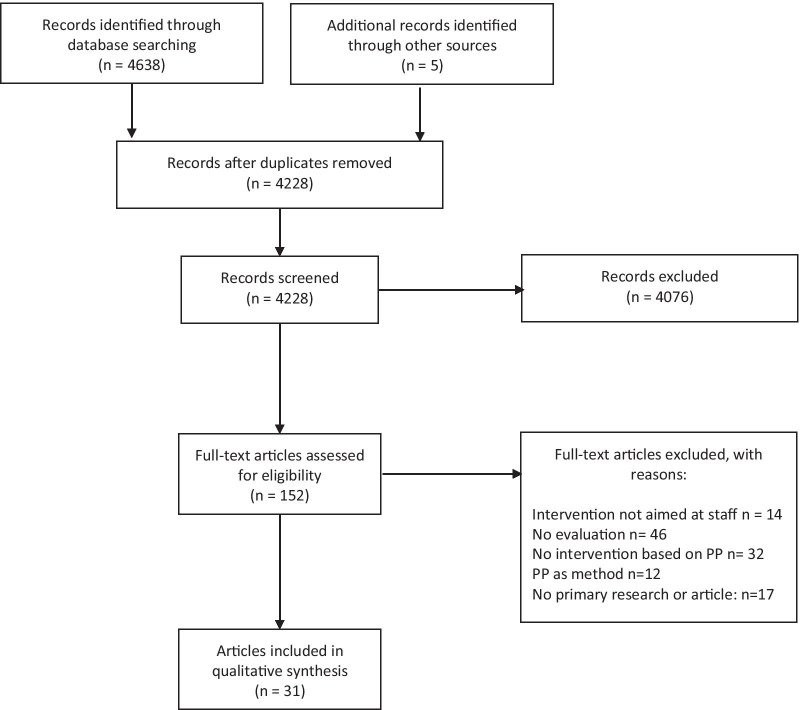
PRISMA flow diagram depicting the results of the literature search

Our search retrieved 4638 articles and identified another five articles through reference screening of included articles, as shown in Fig. 2. After removal of duplicates, 4228 articles were screened for title and abstract. We screened 152 full-text articles for eligibility and included 29 studies (31 articles). Articles were excluded at full-text screening for the following reasons: no evaluation of the intervention, (*n* = 46), no positive psychology (*n* = 32), no primary research (*n* = 17), intervention not aimed at health personnel (*n* = 14) and positive psychology as research method instead of intervention (*n* = 12).

### Quality appraisal

Most articles were of low quality (*n* = 19), six articles were of medium quality and six were deemed to be of high quality (Additional file 2: Quality appraisal). Articles scored particularly low on methods as there was little explanation about evaluation methods and reasons for choosing these methods.

### Overview of included studies

An overview of extracted data is presented in Additional file 3: Overview of included studies. Most studies were published since 2014 (*n* = 15). Only one article was published before 2007. Eight studies were conducted in the UK and seven in the USA. Others were performed in Canada (*n* = 3), India (*n* = 2), Australia (*n* = 2), Belgium, China, Denmark and The Netherlands (*n* = 1 each). The majority of the interventions were implemented in secondary or higher, care settings (*n* = 21), while other settings included an autism care organisation (*n* = 3), care homes (*n* = 2) and primary care settings (*n* = 2).

A wide variety of health personnel, working in various wards and at different levels, were included in the interventions. The majority of studies included nurses or nursing managers (*n* = 16). Allied health professions were included in nine studies and medical doctors in six. In four studies it was not clarified which professions were included. The exact place or whereabouts of the implementation of the intervention was rarely described. The most common department for implementation of an intervention was the emergency department (*n* = 3). Other departments included gynaecology (*n *= 1), internal medicine (*n* = 1), radiology (*n* = 1) and surgery (*n* = 1).

We identified eight different types of interventions: appreciative inquiry interventions (*n* = 12), staff training programmes (*n* = 5), coaching programmes (*n* = 4), a video feedback intervention (*n* = 3), a workers’ health surveillance module (*n* = 1), Strengthscope™ (*n* = 1), PROPEL (*n* = 1) and an excellence reporting intervention (*n* = 1). All interventions, except the workers’ health surveillance module and the excellence reporting intervention, were delivered face-to-face. The duration and number of sessions of the interventions varied widely. For example for AI, one organisation held two sessions of 4.5 h each [29], while in another three full-day sessions were held [30, 31].

The rationale behind the chosen interventions was their positive nature, which allowed participants to discover strengths and act as an alternative to negativity or weaknesses (*n* = 13). Additionally, interventions were implemented to: reflect, create new perspectives and improve self-insight (*n* = 5), increase levels of peer support and interaction (*n* = 3), allow participants to be active agents in their own learning process (*n* = 1), help participants to be more compassionate and nurturing with self (*n* = 1), re-establish a sense of direction (*n* = 1), and enhance personal resources (*n* = 1). Finally, interventions were chosen due to the flexibility of intervention, focus on change and to overcome implementation barriers (*n* = 3). There was no association between intervention type and rationale for use.

### Outcomes

We identified 54 different outcomes in the included studies, as presented in Table 1. Organisational performance was not mentioned as an outcome in any of the included studies. A wide variety of approaches to measurement of these outcomes were used across the studies, including self-report, individual in-depth interviews and validated questionnaires (Additional file 3: Overview of included studies). 35 outcomes were assessed quantitatively, 14 were assessed qualitatively and five were assessed both qualitatively and quantitatively. Out of the 40 quantitative outcomes there were seven with solely statistically significant positive outcomes and seven outcomes that were statistically significant in some studies, and neutral in others. Additionally there were 25 outcomes that were neutral in all studies and one outcome that showed a neutral or negative outcome in all studies. Of the 14 qualitatively assessed outcomes, 12 were positive, one was neutral and one was negative.

**Table 1:** Outcomes of interventions

		Intervention														Total			Total		
Cluster	Outcome	Appreciative inquiry		Coaching programme		PROPEL		Staff training programme		Strengthscope™		Video feedback intervention		Workers' Health Surveillance Module		QUANT			QUAL		
		QUANT	QUAL	QUANT	QUAL	QUANT	QUAL	QUANT	QUAL	QUANT	QUAL	QUANT	QUAL	QUANT	QUAL	+	0	−	+	0	−
Attitudes as result of work	Job satisfaction	Shendell-Falik (0)				Muha (0)		Guzman1 (0), Siu (+)								1	3				
	Morale		Dewar (+)																1		
	Motivation		Dematteo (+),	Yu (+)												1			1		
	Resilience			Gray(0)				vanAgteren(+)								1	1				
	Trust		Ruhe(+)																1		
	Work Engagement					Muha(0)								Bolier(+)		1	1				
Attrition	Likelihood of leaving	Buck(0)															1				
	Retention					Muha(0)											1				
	Sick leave	Wright(+)*				Muha(0)										1	1				
	Turnover rates	Stefaniak(0)															1				
	Vacancy rates	Challis(0),Wright(0),Stefaniak(0)															3				
Burnout	Anxiety							vanAgteren(0)						Bolier(0)			2				
	Burnout							Siu(+)*,Eastubrg(0)								1	1				
	Depression							vanAgteren(0)						Bolier(0)			2				
	Emotional exhaustion			Palamara(0)													1				
	Stress							vanAgteren(0),Siu(+)*								1	1				
Collaboration at work	Appreciation of others										Macafee(+)								1		
	Awareness of interactions												James1(+)						1		
	Common ground	Ruhe(+)														1					
	Connection to others		Ruhe(+),Dewar(+),Dematteo(+)																3		
	Interaction and support	Bergs(+)	Dewar(+)					Guzman1(0/+)								2	1		1		
	Peer cohesion							Eastburg(0)									1				
	Sense of community	Buck(0)															1				
	Team working	Sharma(+)							Guzman(+)							1			1		
Confidence	Confidence		Dewar(+)								Macafee(+)		Hall(+)						3		
	Personal accomplishment				Palamara(0)															1	
	Self-realisation				Ammentorp(+)														1		
	Sense of competence							Guzman1(0)									1				
Information	Quality of information	Bergs(0)															1				
	Relevance of information	Bergs(0)															1				
Knowledge and personal skills	Knowledge		Dewar(+)													1			1		
	Learning transfer							Guzman1(0/-)									1	1			
	Insight		Ruhe(+)	Yu(+)	Ammentorp(+)								James(+)			1			3		
	Self-reflection			Yu(0)							Macafee(+/−)		James(+)			1			2		1
Positive emotions	Celebration		Dewar(+)																1		
	Fun								Guzman(+)										1		
	Happiness							Guzman1(0)									1				
	Positive mental health							Siu(+)						Bolier(+)		2					
	Well-being			Gray(+),Yu(0)				Austin(0),Guzman(0),vanAgteren(0)						Bolier(0)		1	5				
Service user perspective	Patient satisfaction	Shendell-Falik(0)				Muha(0)											2				
	Service user perspective												James(+),James1(+)						2		
Supervision and management	Management processes	Sharma(0)															1				
	Manager satisfaction	Stefaniak(0)															1				
	Supervisor support							Eastburg(0)									1				
Work practices	Approaches to dementia							Guzman1(0)									1				
	Behaviour change		Dematteo(+)																1		
	Cleanliness	Sharma(0)															1				
	Compliance with guidelines	Shendell-Falik(0)															1				
	Infection control practices		Sharma(0)																	1	
	Nutritional assessment	Shendell-Falik(0)															1				
	Preventive service delivery	Ruhe(0)															1				
	Proactivity			Yu(+)												1					
	Puerperal infection	Hussein(0)															1				
	Sustainable change		Dematteo(−)																		1
Total	+	3	12	4	1	0	0	7	2	3	0	0	6	2	2	18			25		
	0	18	0	4	1	5	0	15	0	0	0	0	0	3	3		43			2	
	-	0	1	0	0	0	0	1	0	1	0	0	0	0	0			1			2

The most commonly mentioned outcome was well-being, which was mentioned in six studies, but never measured in the same way. Well-being showed a statistically significant improvement in only one article, the others showed neither improvement nor worsening. The interventions including well-being as outcome were a coaching programme (*n* = 2), a staff training programme (*n* = 3) and the ‘Workers Health Surveillance Module’. ‘Positive mental health’ improved significantly in two interventions, the Workers’ Health Surveillance module and a staff training programme.

‘Interaction and support’, which also showed improvement in a qualitative article, statistically improved in AI interventions (*n* = 2), and a staff training programme. Other outcomes that showed statistically significant improvement, in one article each, were burnout, job satisfaction (in one of the four articles that assessed it), insight, motivation, proactivity, resilience, sick leave and work engagement. Additionally, insight, motivation and resilience also showed improvement in qualitative evaluations.

Self-reflection was assessed in three studies, one quantitative and two qualitative, looking at a coaching programme, a staff training programme and PROPEL. There were no statistically significant outcomes, but the qualitative article mentioned improvement. None of the articles looking at vacancy rates showed a significant change. Three articles, assessing AI, mentioned ‘improved connection to others’. Anxiety and depression were both assessed by two articles, but neither showed a statistically significant improvement.

Three outcomes showed a deterioration after the intervention: learning transfer, showing reduced readiness for transferring learning into practice (statistically significant), self-reflection, showing less self-reflection following the intervention (not statistically significant) and sustainable change (not statistically significant).

### Mechanisms

We identified 49 different mechanisms as described in the included articles (see Additional file 4: Overview of identified mechanisms). While most mechanisms were only mentioned by one article (*n* = 41), some were mentioned by multiple. ‘Recognising and reframing negative interpretations’ was mentioned in five articles assessing a coaching programme (*n* = 1), AI (*n* = 3) and an excellence reporting intervention (*n* = 1). ‘Sharing experiences and history’ and ‘time to reflect’ were both mentioned by four articles, assessing AI (*n* = 4), and a coaching programme (*n* = 1), AI (*n* = 1) and video interaction guidance (*n* = 2), respectively. ‘Increased awareness’ was mentioned in three articles assessing video interaction guidance (*n* = 2) and AI (*n* = 1). Finally, ‘sense of community’, ‘recognition of experts in own context and as members of team’, ‘focus on creativity, mutual respect and relationship building’, and ‘breaking down interprofessional hierarchies’ were each mentioned by two articles.

### Contextual factors

We identified 24 contextual factors of which seven were mentioned in more than one article (see Additional file 5: Overview of identified contextual factors). There were seven factors that acted as barriers, while the other 17 acted as facilitators. The facilitator that was most often mentioned, in eight articles, was ‘managerial support’. Other frequently mentioned facilitating contextual factors were ‘no professional relationship between coach/trainer and participant’ and ‘positivity welcome contrast to problem-based approach’, which were both mentioned by three studies. ‘Champion commitment’ and ‘online interventions that are accessible and affordable’ were facilitating contextual factors mentioned by two studies. Finally, three studies mentioned ‘stressful work environments’ and ‘history of failed interventions’ as barriers to implementation. Contextual factors were split into three categories relating to timing, as described in the methods section, and shown in Table 2.

**Table 2:** Overview of contextual factors

Category	Contextual factor
Before design and implementation of intervention	Trust in management Sufficiently high morale Sufficient human resources Personnel receptive to change Perceived need for intervention Human potential maximised in organisation Past successes
During design and initial implementation of intervention	Leadership buy-in Clear communication about intervention Buy-in from participants Champion commitment Safe environment for intervention Nature of intervention doable with existing time pressures Nature of intervention aligns with personality participants Support from external organisation
During intervention	Compassionate, authentic and optimistic coach/trainer Adequate durations of sessions and session intervals Sufficient access to intervention Reminders to those who have not yet participated Regular reinforcement during training Diversity encouraged

### Logic model

**Fig 3:**
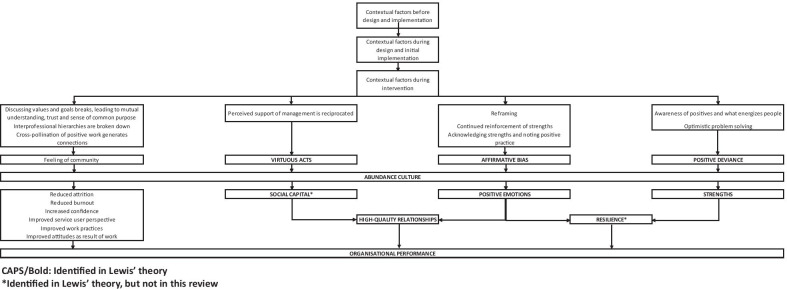
Theory explaining impact of positive psychology interventions on organisational performance for healthcare

The developed logic model is presented in Fig. 3, which is the integration of Lewis’ theory with the results of this review as described above. Figure 3 is based on Lewis’ theory, but specific for PP interventions in healthcare organisations. The figure is based on available evidence and, unlike Fig. 1, includes contextual factors as well as an additional mechanisms and intermediate outcomes. With the help of the logic model, we aimed to provide a more simplified overview of our findings as identified outcomes as well as mechanisms varied widely among the included studies.

Contextual factors were added at the top of the logic model as they act as intervention inputs. To simplify, the model contextual factors were listed as category. The contents of each category can be found in Table 2.

Once contextual inputs were identified, we looked at the mechanisms, in Fig. 3 called intervention processes, which explain how the intervention leads to an abundance culture, through which outcomes are achieved. There were three main mechanisms present in Lewis’ theory: virtuous acts, affirmative bias and positive deviance. We identified processes that link the contextual inputs to the realisation of these three mechanisms. For example, reframing from negatives to positives supports affirmative bias. Additionally, a focus on optimistic problem-solving feeds into positive deviance. We also identified a fourth mechanism, the feeling of community. The processes that feed into the feeling of community are the breakdown of interprofessional hierarchies and discussing values and goals with colleagues.

Through the abundance culture, post-immediate outcomes are created, which are expected to lead to improved organisational performance. We included clustered outcomes, as presented in Table 1, which had at least one positive quantitative or qualitative outcome. Lewis’ theory included five outcomes: social capital, positive emotions, strengths, high-quality relationships and resilience. We did not identify social capital or resilience, possibly due to absence of evidence. As they were part of the original theory, we did include them in Fig. 3. We did identify positive emotions, strengths (improved knowledge and personal skills), and high-quality relationships (improved collaboration at work). Other examples of outcomes were reduced attrition, reduced burnout, increased confidence and increased positive emotions.

## Discussion

While organisational performance was not measured in any of the 29 included studies, with the help of Lewis’ theory for impact of PP interventions, we developed a logic model explaining how PP interventions can impact organisational performance in healthcare settings. The recent growth in reported use of PP interventions, with almost half of the included articles published in the last five years, indicates a need for understanding of how and why these interventions could be effective, and what potential facilitators and barriers implementers should be aware of; a need which we have aimed to address in this study. We included both quantitative and qualitative studies in our systematic review, to provide an overview of intended outcomes and perceptions of the evaluated interventions. Interventions were mainly implemented in secondary or higher services in high-income countries, although two of the included studies were conducted in India and China [30–32].

While the logic model is a simplified version of reality, we believe it contains a wealth of information for those developing, implementing and evaluating PP interventions in healthcare settings. For example, to aid the identification of contextual factors that are important to consider before and during design as well as during a PP implementation; and to guide monitoring and evaluation of the impact of PP interventions in healthcare organisations with the many potentially measurable intermediate outcomes that we have identified. Furthermore, our logic model can guide future research, including study methodology, into the impact of PP on organisational performance in healthcare organisations, for example by providing a list of mechanisms to look for. The logic model is generalised, rather than being specific to a particular setting. Implementers should therefore consider the elements of the model alongside their own knowledge of their local setting when using it to guide design, implementation and evaluation of PP interventions.

Because interventions and outcome measures varied widely, it was difficult to compare them. Due to small samples sizes and low quality of included studies, it was difficult to determine the impact of PP interventions on health personnel. Less than half (*n* = 14) of the quantitatively assessed outcomes showed positive statistical significance in at least some of the articles including these outcomes. Additionally, there were only two outcomes that showed statistically significant improvement in more than one article: ‘interaction and support’ [29, 33] and ‘positive mental health’ [32, 34]. It is noteworthy that qualitatively assessed findings were more likely to be positive than quantitatively assessed ones.

The mechanisms we identified also varied and only a few were mentioned in more than one study, although this does not imply that they were not present in studies where they were not reported. The most commonly mentioned mechanism was ‘recognising and reframing negative interpretations’[35–39], which seems important in healthcare, where there is a lot of emphasis on negativity as preventing adverse events is often prioritised. Two mechanisms were mentioned in four articles: ‘sharing experiences and history’ [37, 39–41] and ‘time to reflect’ [35, 39, 42, 43]. The PP interventions brought people from different wards, departments and jobs together, which helped break down barriers and allowed staff to learn from each other. In the day-to-day job of health professionals there seems to be little time to reflect upon practices, particularly on what went well [44, 45], whereas the PP interventions created time to reflect, which was valued.

We identified several contextual factors that are important for achieving impact of a PP intervention. It was considered important that the coach or trainer facilitating the intervention had no professional relationship with the participants [35]. Interventions were hampered by stressful work environments [36], which are common in healthcare, and a history of failed projects [36]. In some studies scepticism regarding the intervention was mentioned as there was uncertainty if momentum could be sustained or if there would be sufficient support from management to keep the intervention going [39, 46]. Lack of support means the intervention is not being used or not being taken forward, which is a demotivating factor for health personnel.

Our study has several limitations. Only a few mechanisms were described in the included studies, which leads us to believe there are additional hidden mechanisms. Additionally, due to wide variation in impact assessments in the included studies, as well as the interventions themselves, we were unable to compare findings across different settings. Moreover, we did not check grey literature, where there may be relevant articles regarding evaluations of interventions using PP. Furthermore, the quality of included articles is low, in particular the methods used for evaluation of interventions were of substandard quality, making it difficult to determine the actual impact of the assessed interventions.

## Conclusion

Now, more than ever, new strategies are needed to support and retain the health workforce. Our systematic review has shown that while outcomes of PP intervention for health personnel varied widely, and few outcomes were statistically significant, possibly due to absence of evidence, trends in both the quantitative articles and the qualitative outcomes show that positive mental health, interaction and support and well-being of health personnel increased through participation in PP interventions. Additionally, we developed a logic model explaining how PP interventions can impact organisational performance, as well as intermediate outcomes, including well-being, of healthcare personnel. This logic model could support designers, implementers and evaluators of PP interventions in healthcare. However, more research about the impact of interventions using positive psychology is needed, in particular to determine impact quantitatively. Additionally, future research should focus on making mechanisms of interventions using PP more explicit.

## Supplementary Information

**Additional file 1.** Search strategy.

**Additional file 2.** Quality appraisal.

**Additional file 3.** Overview of included articles.

**Additional file 4.** Overview of identified mechanisms.

**Additional file 5.** Overview of identified contextual factors.

## Data Availability

The datasets used and/or analysed during the current study are available from the corresponding author on reasonable request.

## Abbreviations

AI: Appreciative inquiry
ED: Emergency Department
ICU: Intensive Care Unit
LPN: Licences Practical Nurse
M: Mean
UK: United Kingdom
Qual: Qualitative
Quant: Quantitative
RN: Registered nurse
SD: Standard deviation
SPANE: Scale of Positive and Negative Experience
VIG: Video interaction guidance
