# Supplementary material for: Outcomes, mechanisms and contextual factors of positive psychology interventions for health workers: a systematic review of global evidence
Source: Hum Resour Health. 2021 Feb 27;19:24. doi: 10.1186/s12960-021-00564-5 (PMC7910793; doi:10.1186/s12960-021-00564-5)
Supplement: Supplementary file 1 — Additional file 1. Search strategy. [file 12960_2021_564_MOESM1_ESM.docx]

**Additional file 2_Search Strategy**

Psychinfo - 433

| 1. | exp Health Personnel/ |
| --- | --- |
| 2. | (allergist* or allied health personnel or anatomist* or anesthetist* or anesthesiologist* or cardiologist* or dentist* or dermatologist* or doctor* or emergency medical technician* or emergency physician or endocrinologist* or family physician* or gastroenterologist* or general practitioner* or geriatrician* or gerontologist* or gyneaecologist* or gynecologist* or health personnel or hematologist* or hospitalist* or immunologist* or intensivist* or internist* or medical faculty or medical staff or midwi* or neonatologist* or nephrologist* or neurologist* or neurosurgeon* or nurse* or nursing faculty or nutritionist* or obstetrician* or occupational health physician* or occupational therapist* or oncologist* or ophtalmologist* or optometrist* or orthopedic surgeon* or otolaryngologist* or paediatrician* or pathologist* or pharmacist* or pharmacy technician* or physical therapist* or physician* or podiatrist or primary care physician* or psychiatrist* or pulmonologist* or radiologist* or rheumatologist* or surgeon* or urologist* or women physician*) |
| 3. | (basic health worker* or birth attendant* or community health agent* or community health aide* or community health assistant* or community health extension worker* or community health nurse* or community health officer* or community health promotor* or community health surveyor* or community health volunteer* or community health worker* or community-based practitioner* or family health worker* or frontline health worker* or health auxiliar* or health extension worker* or lady health worker* or lay health worker* or traditional birth attendant* or village health volunteer* or village health worker* or volunteer health worker*) |
| 4. | 1 or 2 or 3 |
| 5. | (appreciative inquiry or 4d cycle) |
| 6. | Positive psychology/ |
| 7. | (strength-based or strength*) adj4 coaching |
| 8. | positiv* adj4 coaching |
| 9. | positiv* adj4 feedback |
| 10. | excellen* adj4 feedback |
| 11. | (strength-based or strength*) adj4 feedback |
| 12. | 5 or 6 or 7 or 8 or 9 or 10 or 11 |
| 13. | 4 and 12 |
| 14 | Limit 13 to English language |

Embase - 2795

| 1. | exp health care personnel/ |
| --- | --- |
| 2. | (allergist* or allied health personnel or anatomist* or anesthetist* or anesthesiologist* or cardiologist* or dentist* or dermatologist* or doctor* or emergency medical technician* or emergency physician or endocrinologist* or family physician* or gastroenterologist* or general practitioner* or geriatrician* or gerontologist* or gyneaecologist* or gynecologist* or health personnel or hematologist* or hospitalist* or immunologist* or intensivist* or internist* or medical faculty or medical staff or midwi* or neonatologist* or nephrologist* or neurologist* or neurosurgeon* or nurse* or nursing faculty or nutritionist* or obstetrician* or occupational health physician* or occupational therapist* or oncologist* or ophtalmologist* or optometrist* or orthopedic surgeon* or otolaryngologist* or paediatrician* or pathologist* or pharmacist* or pharmacy technician* or physical therapist* or physician* or podiatrist or primary care physician* or psychiatrist* or pulmonologist* or radiologist or rheumatologist* or surgeon* or urologist* or women physician*) |
| 3. | (Basic health worker* or birth attendant* or community health agent* or community health aide* or community health assistant* or community health extension worker* or community health nurse* or community health officer* or community health promotor* or community health surveyor* or community health volunteer* or community health worker* or community-based practitioner* or family health worker* or frontline health worker* or health auxiliar* or health extension worker* or lady health worker* or lay health worker* or traditional birth attendant* or village health volunteer* or village health worker* or volunteer health worker*) |
| 4. | 1 or 2 or 3 |
| 5. | (appreciative inquiry or 4d cycle) |
| 6. | Positive psychology |
| 7. | (strength-based or strength*) adj4 coaching |
| 8. | positiv* adj4 coaching |
| 9. | positiv* adj4 feedback |
| 10. | excellen* adj4 feedback |
| 11. | (strength-based or strength*) adj4 feedback |
| 12. | 5 or 6 or 7 or 8 or 9 or 10 or 11 |
| 13. | 4 and 12 |
| 14 | Limit 13 to English language |

Medline - 1058

| 1. | exp Health Occupations/ |
| --- | --- |
| 2. | exp Health Personnel/ |
| 3. | (Basic health worker* or birth attendant* or community health agent* or community health aide* or community health assistant* or community health extension worker* or community health nurse* or community health officer* or community health promotor* or community health surveyor* or community health volunteer* or community health worker* or community-based practitioner* or family health worker* or frontline health worker* or health auxiliar* or health extension worker* or lady health worker* or lay health worker* or traditional birth attendant* or village health volunteer* or village health worker* or volunteer health worker*) |
| 4. | (allergist* or allied health personnel or anatomist* or anesthetist* or anesthesiologist* or cardiologist* or dentist* or dermatologist* or doctor* or emergency medical technician* or emergency physician or endocrinologist* or family physician* or gastroenterologist* or general practitioner* or geriatrician* or gerontologist* or gyneaecologist* or gynecologist* or health personnel or hematologist* or hospitalist* or immunologist* or intensivist* or internist* or medical faculty or medical staff or midwi* or neonatologist* or nephrologist* or neurologist* or neurosurgeon* or nurse* or nursing faculty or nutritionist* or obstetrician* or occupational health physician* or occupational therapist* or oncologist* or ophtalmologist* or optometrist* or orthopedic surgeon* or otolaryngologist* or paediatrician* or pathologist* or pharmacist* or pharmacy technician* or physical therapist* or physician* or podiatrist or primary care physician* or psychiatrist* or pulmonologist* or radiologist* or rheumatologist* or surgeon* or urologist* or women physician*) |
| 5. | 1 or 2 or 3 or 4 |
| 6. | (appreciative inquiry or 4d cycle) |
| 7. | Positive psychology |
| 8. | (strength-based or strength*) adj4 coaching |
| 9. | (strength-based OR strength*) adj4 feedback |
| 10. | positiv* adj4 coaching |
| 11. | positiv* adj4 feedback |
| 12. | excellen* adj4 feedback |
| 13. | 6 or 7 or 8 or 9 or 10 or 11 or 12 |
| 14. | 5 and 13 |
| 15. | Limit 14 to English language |

Scopus - 1605

TITLE-ABS-KEY ( ( ( "health personnel" OR "health occupation" OR "allergist*" OR "allied health personnel" OR "anatomist*" OR "anesthetist*" OR "anesthesiologist*" OR "cardiologist*" OR "dentist*" OR "dermatologist*" OR "doctor*" OR "emergency medical technician*" OR "emergency physician*" OR "endocrinologist*" OR "family physician*" OR "gastroenterologist*" OR "general practitioner*" OR "geriatrician*" OR "gerontologist*" OR "gynaecologist*" OR "gynecologist*" OR "hematologist*" OR "hospitalist*" OR "immunologist*" OR "intensivist*" OR "internist*" OR "medical faculty" OR "medical staff" OR "midwi*" OR "neonatologist*" OR "nephrologist*" OR "neurologist*" OR "neurosurgeon*" OR "nurse*" OR "nursing faculty" OR "nutritionist*" OR "obstetrician*" OR "occupational health physician*" OR "occupational therapist*" OR "oncologist*" OR "ophtalmologist*" OR "optometrist*" OR "orthopedic surgeon*" OR "otolaryngologist*" OR "paediatrician*" OR "pathologist*" OR "pharmacist*" OR "pharmacy technician*" OR "physical therapist*" OR "physician*" OR "podiatrist*" OR "primary care physician*" OR "psychiatrist*" OR "pulmonologist*" OR "radiologist" OR "rheumatologist*" OR "surgeon*" OR "urologist*" OR "women physician*" ) OR ( "basic health worker*" OR "birth attendant*" OR "community health agent*" OR "community health aide*" OR "community health assistant*" OR "community health extension worker*" OR "community health nurse*" OR "community health officer*" OR "community health promotor*" OR "community health surveyor*" OR "community health volunteer*" OR "community health worker*" OR "community-based practitioner*" OR "family health worker*" OR "frontline health worker*" OR "health auxiliar*" OR "health extension worker*" OR "lady health worker*" OR "lay health worker*" OR "traditional birth attendant*" OR "village health volunteer*" OR "village health worker*" OR "volunteer health worker*" ) ) AND ( ( "appreciative inquiry" OR "4D cycle" ) OR ( "positive psychology" ) OR ( ( "strength-based" OR "strengths" ) W/4 coaching ) OR (( "strength-based" OR "strengths" ) W/4 feedback) OR ( "excellen*" W/4 feedback ) OR ( "positiv*" W/4 coaching ) OR ( "positiv*" W/4 feedback ) ) )

CINAHL - 1058

| 1. | ( ( "health personnel" OR "health occupation" OR "allergist*" OR "allied health personnel" OR "anatomist*" OR "anesthetist*" OR "anesthesiologist*" OR "cardiologist*" OR "dentist*" OR "dermatologist*" OR "doctor*" OR "emergency medical technician*" OR "emergency physician*" OR "endocrinologist*" OR "family physician*" OR "gastroenterologist*" OR "general practitioner*" OR "geriatrician*" OR "gerontologist*" OR "gynaecologist*" OR "gynecologist*" OR "hematologist*" OR "hospitalist*" OR "immunologist*" OR "intensivist*" OR "internist*" OR "medical faculty" OR "medical staff" OR "midwi*" OR "neonatologist*" OR "nephrologist*" OR "neurologist*" OR "neurosurgeon*" OR "nurse*" OR "nursing faculty" OR "nutritionist*" OR "obstetrician*" OR "occupational health physician*" OR "occupational therapist*" OR "oncologist*" OR "ophtalmologist*" OR "optometrist*" OR "orthopedic surgeon*" OR "otolaryngologist*" OR "paediatrician*" OR "pathologist*" OR "pharmacist*" OR "pharmacy technician*" OR "physical therapist*" OR "physician*" OR "podiatrist*" OR "primary care physician*" OR "psychiatrist*" OR "pulmonologist*" OR "radiologist" OR "rheumatologist*" OR "surgeon*" OR "urologist*" OR "women physician*" ) OR ( "basic health worker*" OR "birth attendant*" OR "community health agent*" OR "community health aide*" OR "community health assistant*" OR "community health extension worker*" OR "community health nurse*" OR "community health officer*" OR "community health promotor*" OR "community health surveyor*" OR "community health volunteer*" OR "community health worker*" OR "community-based practitioner*" OR "family health worker*" OR "frontline health worker*" OR "health auxiliar*" OR "health extension worker*" OR "lady health worker*" OR "lay health worker*" OR "traditional birth attendant*" OR "village health volunteer*" OR "village health worker*" OR "volunteer health worker*" ) ) |
| --- | --- |
| 2. | (MH "Health Personnel+") |
| 3. | (MH "Health Occupations+") |
| 4. | S1 OR S2 OR S3 |
| 5. | "positive psychology" |
| 6. | "appreciative inquiry" |
| 7. | (strength-based OR strength*) n4 coaching |
| 8. | positiv* n4 coaching |
| 9. | positiv* n4 feedback |
| 10. | excellen* n4 feedback |
| 11. | (strength-based OR strength*) n4 feedback |
| 12. | S5 OR S6 OR S7 OR S8 OR S9 OR S10 OR S11 |
| 13. | S4 AND S12 |
